# Opioid Substitution: More than Only Methadone!

**DOI:** 10.1192/j.eurpsy.2022.146

**Published:** 2022-09-01

**Authors:** M. Torrens

**Affiliations:** Hospital del Mar, Universitat Autònoma de Barcelona, Psychiatry, Barcelona, Spain

**Keywords:** buprenorphine, opioid addiction, new synthetic opioids, methadone

## Abstract

Opioid misuse and its rising rates of morbidity and associated mortality is an increasing area of concern worldwide. The licit/illicit consumption of opioids ranging from plant-based substances and pharmaceutical drugs (particularly analgesia) to the new synthetic opioids, has brought opioid use disorder (OUD) back to the public health concerns, including not only prevention but also availability of evidence-based treatments. Agonist opioids have demonstrated by long high efficacy and effectiveness for OUD treatment. Although methadone has been the more prescribed drug in most of the countries where opioid agonist treatment is available, other agonist opioids can be prescribed. We will present a start of the art of other agonist opioids available for the treatment of OUD, emphasizing in the differences among them, in line with of personalizing treatment in addiction. We will focus on morphine slow release, buprenorphine (with or without naloxone, sublingual or long-lasting) and diacetylmorphine.

**Disclosure:**

MT has been consultant/advisor and/or speaker for Gilead Sciences, Merck Sharp & Dohme Corp, Servier, Adamed, Lundbeck, Camurus, Rovi and Molteni.

